# Marine Antifreeze Proteins: Structure, Function, and Application to Cryopreservation as a Potential Cryoprotectant

**DOI:** 10.3390/md15020027

**Published:** 2017-01-27

**Authors:** Hak Jun Kim, Jun Hyuck Lee, Young Baek Hur, Chang Woo Lee, Sun-Ha Park, Bon-Won Koo

**Affiliations:** 1 Department of Chemistry, Pukyong National University, Busan 48513, Korea; 89guti14@gmail.com; 2 Unit of Polar Genomics, Korea Polar Research Institute, Incheon 21990, Korea; justay@kopri.re.kr (C.W.L.); psh@kopri.re.kr (S.-H.P.); 3 Tidal Flat Research Institute, National Fisheries Research and Development Institute, Gunsan, Jeonbuk 54014, Korea; hur0100@korea.kr

**Keywords:** antifreeze proteins, ice-binding proteins, ice recrystallization inhibition, cryoprotectant, slow-freezing, vitrification

## Abstract

Antifreeze proteins (AFPs) are biological antifreezes with unique properties, including thermal hysteresis (TH), ice recrystallization inhibition (IRI), and interaction with membranes and/or membrane proteins. These properties have been utilized in the preservation of biological samples at low temperatures. Here, we review the structure and function of marine-derived AFPs, including moderately active fish AFPs and hyperactive polar AFPs. We also survey previous and current reports of cryopreservation using AFPs. Cryopreserved biological samples are relatively diverse ranging from diatoms and reproductive cells to embryos and organs. Cryopreserved biological samples mainly originate from mammals. Most cryopreservation trials using marine-derived AFPs have demonstrated that addition of AFPs can improve post-thaw viability regardless of freezing method (slow-freezing or vitrification), storage temperature, and types of biological sample type.

## 1. Introduction

Antifreeze proteins (AFPs) are biological antifreeze materials originally found in polar fish; AFPs can bind to ice and subsequently inhibit the growth of the ice crystals. Fish can inhabit ice-laden or cold seawater below the freezing point (−0.7 °C) of their blood serum by virtue of AFPs [1,2,3,4]. However, in a literal sense, the term AFP is a misnomer since AFP does not stop freezing of the blood serum or solution containing AFP. Hence, the term ice-binding protein (IBP) has been proposed to include any protein that binds to ice including AFPs [5]. The term IBP has a bit more nuance than the term AFP. The term ice structuring protein (ISP), which is not used frequently, is synonymous with AFP. However, AFPs are a subset of the larger class of IBPs that includes ice nucleating proteins. In short, all AFPs are IBPs, but not all IBPs are AFPs. In this review, the terms AFP and IBP are used interchangeably.

**Figure 1:**
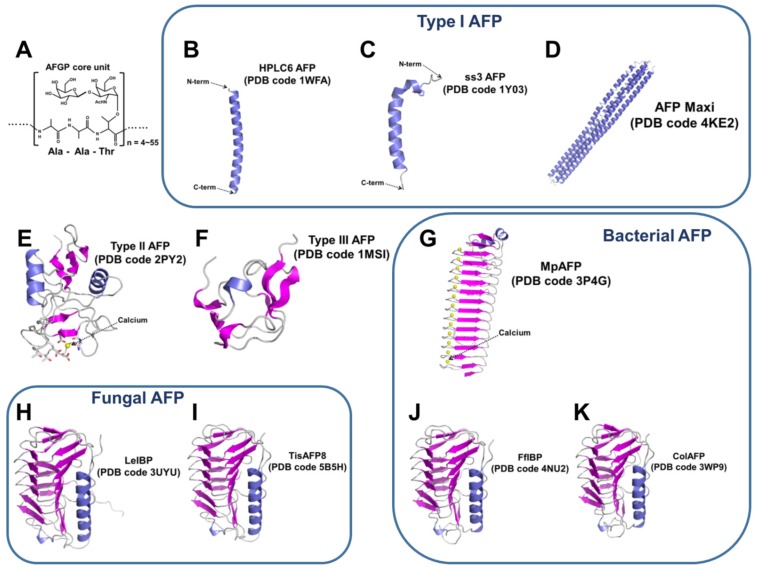
Structural diversity of AFPs: (**A**) core unit structure of antifreeze glycoproteins (AFGPs); (**B**) Type I HPLC6 AFP structure; (**C**) Type I ss3 AFP structure; (**D**) the structure of AFP Maxi from winter flounder, *Pseudopleuronectes americanus*; (**E**) calcium-dependent type II AFP structure; (**F**) Type III HPLC12 AFP structure; (**G**) the structure of MpAFP from *Marinomonas primoryensis*; (**H**) the structure of LeIBP from *Glaciozyma* sp. AY30; (**I**) the structure of TisAFP8 from *Typhula ishikariensis*; (**J**) the structure of FfIBP from *Flavobacterium frigoris* PS I; and (**K**) the structure of ColAFP from *Colwellia* sp. strain SLW05.

Marine organisms known to possess or express AFPs, as shown in Figure 1, include bacteria [6,7,8,9], fungi [10,11,12], crustacean [13], microalgae [14,15,16,17,18,19], and fish [20]. Propelled by next-generation sequencing (NGS) technologies, identification of antifreeze genes from marine organisms has advanced rapidly within the last few years. However, until now, other than fish AFPs, only a few AFPs have been thoroughly characterized from *Colwellia* sp. [21], *Flavobacterium frigoris* [7], *Glaciozyma antarctica* [12,22], *Navicula glaciei* [16], *Fragilariopsis cylindrus* [23,24], and *Chaetocero neoglacile* [15]. The unique function of AFPs, i.e., enabling fish to survive in subfreezing environments, has inspired the researchers in academia and industries to examine the potential applications of AFPs as a potential cryoprotective agents or cryoprotectants (CPAs) in the cryopreservation of biological samples [25,26,27,28,29,30,31]. In this review, we discuss the biophysical and biochemical aspects of marine-derived AFPs as well as investigate past and current research of the practical applications of AFPs in cryopreservation. We also describe the possible role of AFPs in cryopreservation.

## 2. AFP Properties: Thermal Hysteresis (TH), Ice Recrystallization Inhibition (IRI), and Interaction with Biological Membranes

Generally, AFPs can be characterized based on two properties: TH and IRI. However, interaction of AFPs with membranes should not be ruled out. In this section, the unique features of AFPs and their contribution to cryopreservation are discussed briefly (for more an in-depth biophysical discussion on these properties, refer to recent reviews [32,33]).

**Figure 2:**
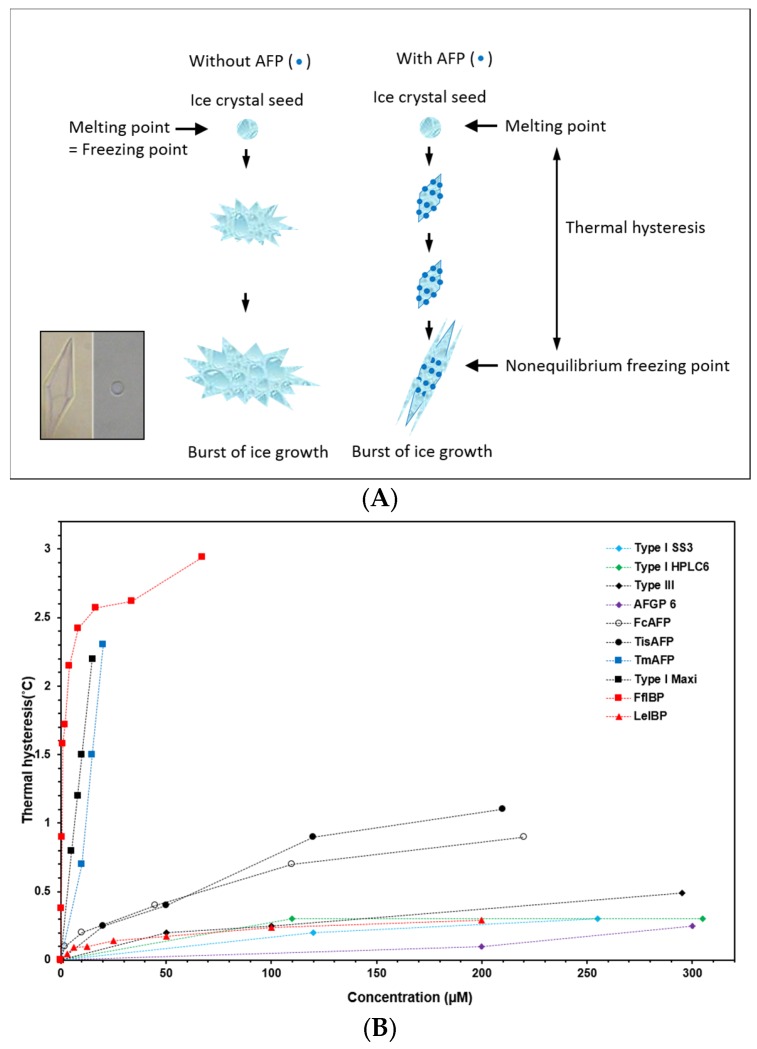
(**A**) Cartoon illustration of TH phenomenon. In the left panel, the ice starts to grow rapidly as temperature drops. However, as shown in the right panel, AFPs adsorb irreversibly on to the specific planes of ice surface, inhibiting the further growth of ice until the temperature reaches nonequilibrium freezing point. This adsorption-inhibition mechanism by AFPs separates melting and freezing points of solution. The inset shows the bipyramidal and lemon ice morphologies created by moderately active type I AFP (left) and hyperactive FfIBP (right), respectively; (**B**) Comparison of TH activities of AFPs from various organisms. TH activity of marine-derived FfIBP (from *Flavobacterium frigoris*), and type I-Hyp (from *Pseudopleuronectes americanus*) are comparable to hyperactive insect and fungal AFPs, TmAFP and TisAFP, respectively, of non-marine origin. Other marine AFPs are moderately. Abbreviations are as follows: TmAFP, *Tenebrio molitor* AFP; TisAFP, *Thyphula ishikariensis* AFP; FcAFP, *Fragilariopsis cylindrus* AFP; and LeIBP, *Glaciozyma* (formerly known as *Leucosporidium*) sp. IBP.

TH refers to the difference between melting and freezing points of a solution. In AFP-containing solution, the temperature gap can be created by irreversible binding of AFPs to ice crystals and subsequent inhibition of their growth until the temperature decreases to the non-equilibrium freezing point [32,34,35,36,37,38,39]. Below the non-equilibrium freezing point, the burst of the ice crystal can be observed (Figure 2A). During the TH gap, AFPs bind to the specific planes of ice crystals, shaping a unique ice morphology. For example, type I AFPs bind to the prism plane of ice and creates a hexagonal bipyramidal shape [35,40], whereas hyperactive FfIBP binds to prism and basal planes and generates a lemon shape [41]. Moderately active AFPs bind to prism and/or pyramidal planes [40,42,43], whereas hyperactive AFPs are able to bind to the basal plane of ice crystals [42,44]. The ice morphology shaped by AFPs is a hallmark of binding of AFPs to ice (inset of Figure 2A) [20]. TH has been used to describe the activity of AFPs quantitatively. For most fish AFPs, the observed TH activity is approximately 1 °C [20,45]. This temperature gap provides enough cushion against seawater during the winter season (−1.9 °C) for polar fish to survive in cold environments. In addition to fish AFPs, many marine AFPs are associated with sea ice [7,13,14,16,21,23,46]. Unlike the blood plasma of polar fish, seawater in brine channels in sea ice undergoes freezing to ice. Hence, AFPs from sea-ice associated bacteria, microalgae, and eukaryotic protists are secreted into the surrounding environment to protect themselves from freezing, and some of them are hyperactive (Figure 2B) [7,21,41].

**Figure 3:**
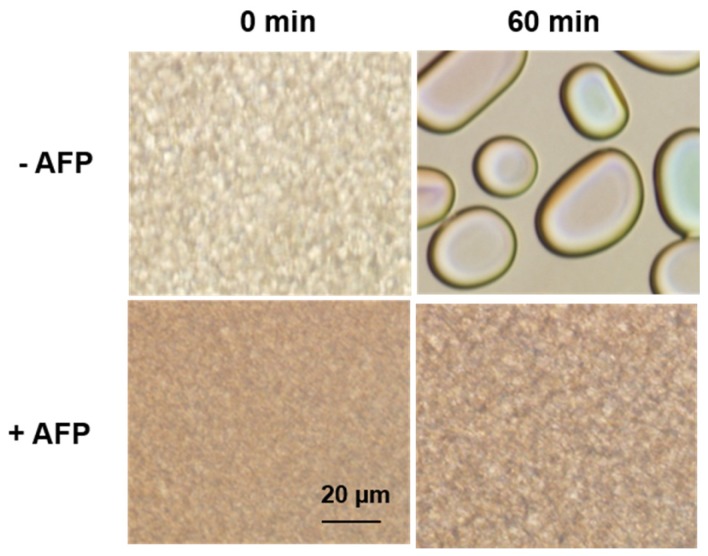
Results of ice recrystallization inhibition (IRI) assay using modified splat assay. In this assay, AFP containing solution was mixed with 30% sucrose in a 1:1 ratio. The mixed solution was spotted between two coverslips and flash frozen. Then, the sample was placed at −6 °C stage and the changes were observed over a specific period of time-in this case 30 min. As in upper panel, larger ice grains grow as expense of smaller ice crystals, while the growth was halted in lower panel in the presence of AFPs. All subfigures are drawn in the same scale.

The second function of AFPs, which may be more useful for cryopreservation, is IRI. Ice recrystallization (IR), as depicted in Figure 3, explains a thermodynamically favorable process in which the formation of larger ice grains takes place at the expense of smaller ones with a high internal energy [47,48]. Eventually, the larger ice crystals formed because of this phenomenon can be fatal to the cryopreserved cells as well as the organisms inhabiting polar or cold regions [49,50]. Fortunately for these organisms, AFPs can inhibit IR at very low concentrations. The AFP-dependent IRI mechanism remains to be elucidated; however, similar to TH activity, IRI is attributed to the binding of AFPs to ice [5,45,51]. AFPs at the interface between the grain boundaries bind to the surface of ice grains and inhibit the growth process [50]. The IRI is more likely to be a key property for cold-tolerant organisms to survive in extremely cold environments [47,52,53,54,55]. To this end, IRI is eventually thought to defend membranes against freezing injury [27,28,29,30,31,56,57]. IRI activity was first analyzed using a splat cooling assay developed by Knight [58]. In splat assays, a small droplet of a solution is expelled from a height of 2 m onto a very cold (−70 °C) metal plate and freezes instantaneously as a polycrystalline wafer. The ice is then annealed at −6 °C over a certain period of time, allowing ice recrystallization to occur. Modified methods have been proposed wherein the ice grains are generated from a few-microliter sample placed between coverslips by flash freezing [54,55] or where the sample inside 10 μL glass capillary undergoes freezing and annealing [59]. However, the IRI result was only semi-quantitatively reported by presenting the IRI endpoint, expressed as mg/mL or μM, where IRI is no longer observed [54,55,58,59]. To assess IRI activity quantitatively, Jackman et al. employed domain recognition software to measure and report the mean grain size (MGS) of the 10 largest ice crystals after the annealing period [60]. This method displayed percent MGS as a function of AFP relative to the control. Very recently, Voets’ group adopted an automated image analysis using the circle Hough transform (CHT) algorithm with a modified splat assay [55] to quantitatively evaluate IRI [61,62]. The CHT is a basic technique for detecting circular objects in a digital image. They attempted to include all ice crystal images instead of only the 10 largest ice crystals in the calculation, which obviously makes the quantitative evaluation of IR kinetics more statistically significant. In this method, the inflection point of the kinetic curve was presented as an IRI endpoint.

**Figure 4:**
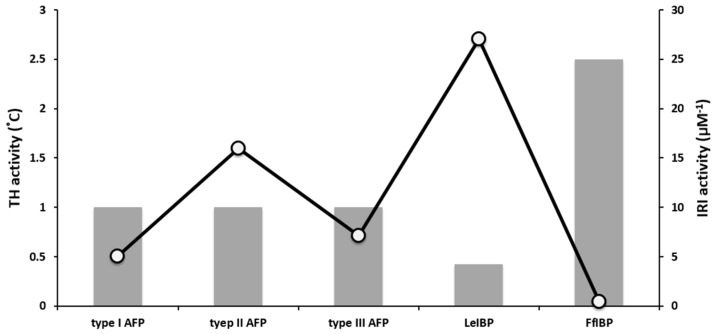
A graph of TH and IRI activities of marine-derived AFPs. TH values, represented as a bar, are from Figure 2B. The IRI activities (O) are expressed as the reciprocal of endpoint of each AFP. The endpoint indicates the lowest concentration at which the AFP shows IRI activity. Higher IRI value means more effective in IRI. The LeIBP is weaker in TH but higher in IRI activity, but vice versa in FfIBP. This plot demonstrates that the TH values are not proportional to IRI activities.

Both TH and IRI properties are based on the affinity of AFPs for ice. Intriguingly, however, TH activity is not always proportional to IRI activity (Figure 4), which remains to be elucidated [63]. In the comparison of TH and IRI activities of hyperactive insect, bacterial, and fish AFPs with moderately active fish AFPs, Yu et al. reported that the TH hyperactivity of AFPs was not reflected in their IRI activity [63]. This was corroborated by other marine-derived AFPs, i.e., LeIBP and FfIBP [7,10,41,44,64]. Olijve et al. also demonstrated that type III AFP and its mutant showed different TH values but almost the same IRI activity [61]. The hyperactive FfIBP showed less activity in IRI, compared to the moderately active LeIBP [7,44]. These results implied that TH activity was not necessarily translated into improvements in the cryopreservation efficiency of biological samples [65,66,67,68,69]. Therefore, the utilization of AFPs in cryopreservation cannot be considered from their TH activity only.

Along with the IRI feature, the interaction of AFPs with membranes (or proteins in membranes) also may also ameliorate the cryoinjury of cells. In the early study of Rubinsky and his colleagues, fish AFPs were found to protect cell membranes during hypothermic storage [70]. As membranes are cooled to low temperatures, one mechanism of injury is often thermotropic phase transition partly due to weakened hydrophobic interactions [71,72,73,74,75]. During the transition from liquid crystalline to gel phase, membranes become leaky, resulting in the loss of intracellular contents [76]. It is not entirely clear what causes leakage during the phase transition; however, this process may be related to defects in packing of the hydrocarbon chains during the coexistence of gel and liquid crystalline domains [71]. Since the phase transition temperature of each lipid depends on the degree of unsaturation of lipid tails and the number of carbons in the lipid alkane chains, model membranes with diverse compositions, such as dielaidoylphosphatidylcholine (DEPC), dielaidoylphos-phatidylethanolamine (DEPE), and dielaidoylphosphatidylglycerol (DEPG), have been used in order to better understand the nature of the interactions between AFPs and cell membranes [72,73,75,77,78,79,80,81,82]. The results showed that these interactions were lipid specific, i.e., the lipid composition of the bilayer dictates whether or not a certain AFP or antifreeze glycoprotein (AFGP) will protect/interact with the membranes [61,62,64,65,66,67,68,69]. Other reports have indicated that the cryoprotective effects of AFPs arose from their interaction with membrane proteins, such as potassium and calcium ion channels [83,84,85,86]. However, in some cases, the addition of AFPs in cryopreservation medium induces leakage from cryopreserved cells [87,88,89,90,91,92,93,94,95]. These results implied that protection against freezing damage by AFPs depends on the type of membrane and the type of AFP [80,96].

## 3. Marine-Derived AFPs

### 3.1. Fish AFPs

Two scientists, Scholander and DeVries, first observed that some fish inhabiting the polar oceans could survive in cold water that occasionally reached sub-zero temperatures [1,3,97]. Following this observation, they attempted to elucidate how these fish could survive in icy water, reaching temperatures below the freezing point of fish blood. When cooling was increased even further, they observed that the growth of ice crystals was sluggish and delayed due to the presence of glycoproteins that depress the noncolligative freezing point of solutions [98]. These proteins were designated AFGPs [3]. Thereafter, nonglycosylated AFPs (type I AFPs) were found in the winter flounder, *Pseudopleuronectes americanus* [99]. In addition, several types of AFPs have been discovered and classified within distinct groups (classified into types I, II, III, and IV) in the Arctic and Antarctic regions. Even though the AFP types are fundamentally different in terms of their primary sequences and three-dimensional structures, they all have equivalent properties allowing them to bind to ice and depress the freezing point of the solutions. Moreover, these different types of AFPs do not seem to share any common ancestor genes.

AFGPs contain a three amino-acid (Ala-Ala-Thr) repeating sequence motif with a disaccharide connected to the hydroxyl group of the threonine residue [100]. However, there are sequence variations at the first residue position; sometimes, the first Ala residue is replaced by a Pro, Thr, or Arg. There are eight AFGPs (AFGP1-8), named according to the number of repeating units. AFGP1 has about 50 repeating units and therefore the highest molecular weight (33.7 kDa), whereas AFGP8 has the lowest molecular weight (2.6 kDa), with only four repeating units. Typically, the antifreeze activities of AFGPs are proportional to the number of repeating units. It is thought that high-molecular-weight AFGPs cover a wider ice surface and inhibit ice growth more efficiently than smaller AFGPs [47,101,102,103,104]. Recent studies have also shown that carbohydrate moieties are important for AFGP activity. Structural studies using nuclear magnetic resonance (NMR) have revealed that carbohydrate moieties and Ala residues are located on opposite sides. This feature confers AFGPs with a helical shape and amphipathic characteristics. Consequently, AFGPs show strong recrystallization properties. However, there are several limitations regarding their commercial utilization toward cryopreservation. Natural polar fish sources are not sufficient to prepare large quantities of AFGPs, and chemical synthesis is difficult to establish in large-scale mass production systems. In contrast, AFPs can be prepared in large quantities by recombinant protein expression techniques. For that reason, AFPs have been more broadly used for application studies than AFGPs. This review focuses on marine AFPs used for cryopreservation applications.

#### 3.1.1. Type I AFPs

Type I AFPs are found in many flounders and sculpins. Type I AFP HPLC6 from winter flounder has been the most extensively studied. This protein possesses 37 amino acids and its sequences are composed of 11 amino acid repeating units [20,105]. Moreover, this protein also has a high Ala residue content, making up 23 of 37 residues. The molecular structure of HPLC6 (PDB code 1WFA) was determined using the X-ray crystallography, which showed that HPLC6 AFP is an α-helical protein with amphipathic characteristics. Another type I AFP has been isolated from the shorthorn sculpin (*Myoxocephalus Scorpius*; ss3 AFP), also displaying a high Ala content (21 Ala residues among a total of 33 residues). The structure of the ss3 AFP (PDB code 1Y03) was determined by NMR spectroscopy. The overall structure of ss3 AFP is similar to that of HPLC6 AFP; however, ss3 AFP contains a Pro residue at position 4, inducing a helix kink. Recently, the four-repeat containing isoform AFP9 and a much larger type I AFP (a 195-residue protein, AFP Maxi) were discovered in winter flounder (*Pseudopleuronectes americanus*). These two proteins exhibit significantly higher TH activities than HPLC6 AFP. Moreover, the increased size of the AFP may induce higher antifreeze activity by facilitating binding to multiple ice crystals and increasing coverage of the ice surface. Furthermore, analysis of the AFP Maxi structure (PDB code 4KE2) revealed that this protein folds into a dimeric four-helix bundle and that its ordered water may be involved in ice binding, thereby enhancing its antifreeze activity.

The ice-binding mechanism of type I AFP was previously investigated through an ice-etching experiment, which is used to identify AFP binding sites. A simple crystal growth and etching technique allows the identification of the crystallographic planes where the binding occurs [40]. Furthermore, ice etching has also been used to identify the ice-binding planes of AFPs and enhanced green fluorescent protein (EGFP) fusion constructs allow their clear visualization. In 1991, Knight et al. reported that type I AFPs from winter flounder (*Pseudopleuronectes americanus*) and Alaskan plaice (*Pleuronectes quadritaberulatus*) adsorb onto the {2 0 −2 1} pyramidal planes of ice, whereas the sculpin (*Myoxocephalus scorpius*) AFP adsorbs onto {2 −1 −1 0}, the secondary prism planes [40]. This finding suggests that each type I AFP has a unique ice-binding mechanism depending on its sequence length and composition. Moreover, ice-binding sites of type I AFPs were analyzed by site-directed mutagenesis, truncated variants, and molecular docking studies [106,107,108,109,110,111]. Currently, it is generally accepted that ice-binding sites of type I AFPs are located on their Ala-rich hydrophobic faces.

#### 3.1.2. Type II AFPs

Type II AFPs are found in sea raven, smelt, herring, and long snout poacher. Type II AFPs are globular cysteine-rich fish AFPs with molecular weights ranging from 11 to 24 kDa. The overall structure of type II AFPs shows numerous similarities with C-type lectin-like domains (CTLDs). Type II AFPs have two α-helices and nine β-strands with specific cysteines forming disulfide bonds. Those disulfide bonds are known for their capacity to increase the structural stability of type II AFPs [112,113,114,115,116]. Structural comparison studies between various groups of type II AFPs showed that even if their amino acid sequence similarity is low, overall, their structures are similar, and they display the same functions. These results indicate that type II AFPs evolved from the backbones of CTLDs [117].

Type II AFPs are distinguished by their dependence on calcium ions to enable their antifreeze activities. Herring and two types of smelts produce Ca^2+^-dependent type II AFPs. Herring type II AFP (hAFP) has close structural similarities with lithostathine (PDB code: 1qdd; root mean square deviation [RMSD] = 1.7 Å for 122 Cα atoms) and mannose-binding protein (PDB code: 1sl6; RMSD = 2.2 Å for 124 Cα atoms). However, these two proteins have no ice-binding activities. Likewise, hAFP has no carbohydrate-binding activity. Thus, this high similarity in carbon backbone structure along with different activities indicates a divergent evolutionary pattern. Another difference between hAFP and C-type lectin protein is the number of cysteine bonds. hAFP has five disulfide bonds, whereas C-type lectin only possesses three or four. Thr96, Leu97, Thr98, and Thr115 residues are important for ice-binding. Interestingly, all of these residues are located near the Ca^2+^ binding site. Therefore, the results obtained from these investigations suggest that Ca^2+^ binding in hAFP is critical for forming an ice-binding state structure and increasing ice-binding activity [115]. Sea raven and long snout poacher produce Ca^2+^-independent type II AFPs. Structural comparisons between Ca^2+^-dependent and -independent type II AFPs showed that several residues near the Ca^2+^ binding site are different. Gln92, Asp94, Glu99, and Asn113 residues of hAFP are substituted with Lys95, Asn97, Asp102, and Asp116 residues, respectively, in long snout poacher AFP (lpAFP). Through these studies, the critical amino acids for Ca^2+^ binding were identified. These amino acids could be important indicators allowing the distinction between Ca^2+^-dependent and-independent type II AFPs [116]. Additionally, a type II AFP was found in Japanese smelt (*Hypomesus nipponensis;* HniAFP), which does not inhabit polar regions, but instead is found in fresh waters in regions near the middle latitudes. Interestingly, HniAFP can bind to Ca*^2^*^+^, but its ice binding activity does not depend on this feature; indeed, despite adding ethylenediaminetetraacetic acid (EDTA) to remove Ca*^2^*^+^, its antifreeze activity was not affected [118].

#### 3.1.3. Type III AFPs

Type III AFPs are small globular proteins with an average molecular weight of 6.5 kDa, found in Antarctic eelepout (*Macrozoarces americanus*) and wolf fish [119,120]. Type III AFPs can be divided into two groups, quaternary-amino-ethyl (QAE) and sulfopropyl (SP) sephadex-binding isoforms, based on both their sequence similarities and affinities for SP and QAEs [121]. QAEs can be further categorized into QAE1 and QAE2 subgroups [122]. According to some studies, QAE1 isoforms have higher TH activities than the QAE2 and SP isoforms. SP and QAE2 isoforms are incapable of stopping ice growth [123,124]. The structures of type III AFPs have been extensively studied, and about 40 models have been solved and deposited in the protein data bank (http://www.rcsb.org/pdb/) to date. Among these, the three-dimensional structure of HPLC12 AFP, belonging to the QAE1 subgroup, was the first to be determined, showing a globular β-sandwich consisting of two antiparallel triple-stranded β-sheets [125,126,127]. Although type III AFPs are mainly composed of several loops, they form stable structures through hydrophobic interactions and a number of hydrogen bonds at the center of the structure. Type III AFPs were found to be active over a broad pH range (2–11), indicating that the protein fold is stable even at extreme pH, which would normally cause protein denaturation [125]. Recent studies have shown that temperature treatment at 80 °C and pressure treatment at 400 MPa (duration of 1 min for both treatments) did not influence the IRI activity of type III AFPs [128]. Interestingly, sialic acid synthase (SAS) has a C-terminal antifreeze-like domain similar to that of type III AFPs. However, these two homologous proteins have very different temperature-dependent stabilities, activities, and backbone dynamics. While type III AFPs are mostly rigid, with a few residues showing slow motions, SAS is remarkably flexible at low temperature [129,130]. These two proteins, displaying different functions, may have evolved from a common structural ancestor.

The most widely accepted hypothesis to describe the mechanism through which type III AFPs interact with ice crystals involves the Thr18 residue located on the flat surface; this residue is thought to be responsible for the recognition and interaction with the primary prism planes of ice. AFPs cover water-accessible ice surfaces, thereby inhibiting ice growth. Several reports have shown that putative ice-binding residues (Gln9, Asn14, Thr15, Ala16, Thr18, and Gln44) are capable of significantly altering TH activity and ice crystal morphology [125,126,131,132]. Notably, the replacement of Thr18 by Asn causes a significant loss of TH activity (90% loss). Computer simulation studies have emphasized that hydrophobic interactions within ice-binding sites are also important for the antifreeze activity of the protein [132,133]. When hydrophobic residues, such as Leu19, Val20, and Val41, were replaced with Ala, a 20% loss in activity was observed. Double mutants (L19A/V41A and L10A/I13A) showed more than 50% loss of activity compared with the activity of the wild-type protein [124]. Ice-etching studies revealed a more complex ice-binding mechanism within type III AFPs, showing that they could interact with both the primary prism and a pyramidal plane of ice [1]. While the QAE1 isoform is able to bind both the primary prism and a pyramidal plane of ice, the SP and QAE2 isoforms can only bind pyramidal ice planes [134]. Interestingly, a triple mutant of the inactive QAE2 isoform (V9Q/V19L/G20V) is able to bind to the primary prism ice plane and shows full TH activity, similar to the QAE1 isoform [135]. More recently, NMR experiments with inactive QAE2-like mutants containing the V20G mutation were reported. These experiments showed that the mutants exhibited increased conformational flexibility and were incapable of binding to the primary prism plane of ice crystals. These results suggested that inactive type III AFPs may be unable to anchor water molecules via H-bond interactions in the first 3_10_ helix (residues 18–22) and therefore have no antifreeze activity [136].

Interestingly, two almost identical type III AFP domains tied by linker residues, designated RD3, were found in nature in the Antarctic eelpout, *Rhigophila dearborni* [137,138,139]. RD3 possesses 5.9-fold higher activity than a single domain in the range of 0 to 0.5 mM. This high activity at low concentrations may be related to the need for much smaller concentrations of AFP for cryopreservation, as mentioned below.

### 3.2. Fungal AFPs

To date, various mushrooms and Basidiomycetous psychrophilic yeast species have been screened and reported to have antifreeze activities. Only two mushrooms (enoki and shiitake), one snow mold fungus (*Typhula ishikariensis*), and two yeast organisms (*Glaciozyma antarctica* and *Glaciozyma* sp. AY30) have been characterized both genomically and for their antifreeze properties [9,10,140,141].

Lee et al. were the first to report the antifreeze activity of a protein isolated from the psychrophilic yeast *Glaciozyma* sp. AY30, itself isolated from an ice core sample of a freshwater pond near the Dasan station, Ny-Ålesund, Svalbard archipelago, Norway, and named LeIBP [10]. LeIBP contains a right-handed β-helical structure, which provides the advantage of a broad-range interaction surface for ice binding [44,64]. The ice-binding site of LeIBP was determined to be a B-face using site-directed mutagenesis experiments [64]. Moreover, the codon-optimized LeIBP (pLeIBP) was constructed and subjected to high-level expression in the *Pichia pastoris* system [142]. In pilot-scale fermentation (700 L), pLeIBP was secreted into culture medium, and the yield was 300 mg/L. The TH activity of pLeIBP was about 0.42 °C, which was similar to that of LeIBP expressed in *E. coli*. The availability of large quantities of pLeIBP allowed us to use this protein in further application studies [65,66,67,143,144,145].

Snow mold fungus (*Typhula ishikariensis*) secretes seven antifreeze protein isoforms composing the TisAFPs [141]. Among them, the structures of TisAFP6 (PDB code 3VN3) and TisAFP8 (PDB code 5B5H) were determined and their antifreeze mechanisms were characterized [146]. The results suggested that TisAFP8 has a more adapted shape and higher hydrophobicity to allow ice binding than TisAFP6, which may possess a higher TH activity. Notably, the overall structures of LeIBP (PDB code 3UYU), TisAFP6, and TisAFP8 are very similar, with RMSD values within 0.73 Å when superimposed.

*Glaciozyma antarctica* AFP (Afp1), described by Hashim et al., possesses both TH and RI activities and shows 30% sequence similarity with TisAFPs [12]. Amino-acid sequence analysis showed that Afp1 contains four α-helices. Shah et al. confirmed the antifreeze activity of each helical peptide [22]. In addition, the NMR structures of the peptides were determined and the ice-binding model was generated using a molecular dynamics method. The results indicated that the Afp1 peptides work like type I AFPs. In 2014, another *Glaciozyma antarctica* AFP (Afp4) was identified and characterized [147]. The Afp4 sequence shows the highest amino acid similarity (93%) to LeIBP. A recombinant Afp4 protein changed ice crystals into hexagonal shaped crystals and showed a TH value of 0.8 °C at a protein concentration of 5 mg/mL.

### 3.3. Diatom AFPs

Studies aiming to identify new AFP genes from polar sea diatoms (*Chaetoceros neogracile*, *Berkeleya* sp., *Navicula* sp., *Fragilariopsis* sp., and *Nitzschia frustulum*) have been performed, and further gene expression studies have shown that the expression of AFP genes is regulated in response to stress conditions, such as cold temperature and high salinity [16,17,23,24,148]. Thus, AFP genes may play an important role in the environmental adaptation of diatoms. In 2009, Gwak et al. first produced recombinant antifreeze protein (Cn-AFP) from a marine diatom, *C. neogracile*, and characterized its antifreeze activity [15]. The TH value of the mature form of Cn-AFP is 0.8 °C, whereas pre-mature Cn-AFP has a 16-fold lower TH activity, indicating that the signal peptide induces improper folding of Cn-AFP or masks the ice-binding site.

### 3.4. Bacterial AFPs

In 2004, Gilbert et al. published an interesting finding showing bacterial AFP screening results obtained from Antarctic lake bacteria [149]. The authors managed to culture 866 bacterial isolates from an Antarctic lake and found RI activity in 19 of these isolates. The first bacterial IBP gene (~25 kDa) was identified, and the protein purified through ice affinity purification, in the sea ice gram-negative bacterium *Colwellia* strain SLW05 [8]. In 2008, other bacterial IBPs (54 kDa) were isolated from a deep Antarctic ice core of the subglacial Lake Vostok, at a depth of 3519 m (GenBank EU694412) [140]. The sequence of the protein is similar to those of IBPs previously found in sea ice habitats, even though the protein is longer. In addition, uncharacterized proteins similar to IBPs were found in sea ice bacteria *Polaribacter irgensii* (ZP_01118128; sequence identity: 61%, sequence similarity: 75%), *Psychromonas ingrahamii* (ZP_01349469; sequence identity: 59%, sequence similarity: 71%), and marine bacterium *Shewanella frididimarina* (YP_749708; sequence identity: 52%, sequence similarity: 69%).

The first bacterial AFP structure was solved using a protein isolated from an Antarctic lake bacterium (*Marinomonas primoryensis;* MpAFP) [150]. MpAFP is a 1.5-MDa protein with calcium-dependent antifreeze activity [6]. The solved MpAFP structure (PDB code 3P4G) shows a calcium-bound β-helical fold and bound water molecules, which fit well onto the ice crystal lattice. Therefore, this structure may explain the anchored clathrate mechanism of AFPs when binding to ice.

Recently, another IBP (FfIBP) from the Antarctic bacterium *F. frigoris* PS1 was identified from sea ice on the shore of McMurdo Sound (GenBank accession no. AHKF00000000.1) and characterized [41,151]. FfIBP shares 56% sequence similarity with LeIBP, but displays an antifreeze activity that is up to 10-fold higher than that of LeIBP. Structural and functional characterization of FfIBP revealed that this protein displays regular motifs (T-A/G-X-T/N motif) and more regularly aligned ice-binding residues on its IBS than LeIBP [7]. These structural differences may confer FfIBP with higher TH activity.

In 2014, structural and biochemical data on an AFP from *Colwellia* sp. strain SLW05 (ColAFP) were published [21]. Interestingly, the ColAFP structure is similar to those of LeIBP, TisAFP, and FfIBP, displaying a β-helical structure. In addition, the alignment of sequences and phylogenetic trees of the bacterial AFPs with those of other AFPs and IBPs suggests that eukaryotic IBPs could have been acquired from bacteria by horizontal gene transfer (HGT) [151]. One theory in favor of HGT is “restricted occurrence”, which suggests that the same small set of organisms can be found in different locations [152]. IBPs seem to satisfy this criterion because hundreds of organisms have IBPs or IBP-like genes. Another potential explanation involves virus-mediated transformation of IBP genes. For example, Arctic cryoconite holes are built on snow, glaciers, or ice caps where viruses are abundant; these viruses are able to infect a broad range of bacterial species and other organisms, suggesting that viruses in the environment may play a role in the exchange of genetic material [153].

Furthermore, a new bacterial AFP [154] with high IRI activity [155] was reported very recently. Metagenomic sequencing of the Antarctic psychrophilic marine ciliate *Euplotes focardii* revealed two sequences encoding IBPs, designated as EFsymbAFP and EFsymbIBP, obtained from its putative bacterial symbiont [154,155]. These IBPs seem to be structurally similar to TisIBP, LeIBP, and FfIBP [154]. Of these, N-terminal 23 residue-deleted EFsymbAFP was recombinantly expressed in *E. coli* and characterized [155]. Its TH activity was 0.53 °C at 50 μM, but its IRI activity was in the nanomolar range, as determined by Voets method. This value is the lowest observed to date. The recombinant protein also effectively protected bacterial cells from freezing damage. Further investigations of this IBP will provide more insight into the relationships among IRI and TH and the evolution of IBP.

## 4. Cryopreservation Using AFPs as a Potential Cryoprotectants (CPAs)

### 4.1. Cryopreservation and Ice Recrystallization

**Figure 5:**
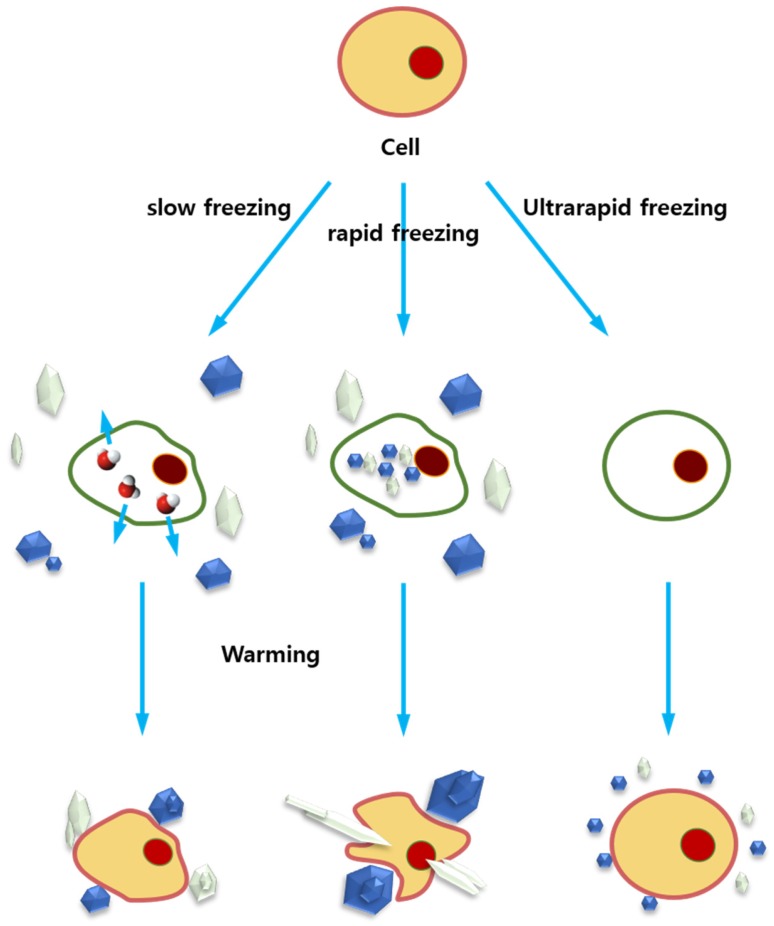
Schematic illustration of freezing rate and ice recrystallization during warming. In slow freezing process, the extracellular ice starts to form below the equilibrium freezing point. Subsequently, water is expelled from inside the cell by osmotic pressure, eventually eliminating the intracellular ice formation. Fast freezing process, however, causes the intracellular ice formation since water cannot leave the cell quickly. In ultrarapid cooling, such as vitrification process, theoretically no bulk ice will form in the presence of higher concentration of CPAs. The ice formed during freezing will become problematic, when the cryopreserved cells are thawed (or warmed). They start to grow bigger: a process known as ice recrystallization. This process is fatal to the cells. Even in vitrification, ice can form during the warming. Therefore, freezing rate should be optimized depending of cell type, CPAs used, etc. The addition of AFPs in freezing media seems to alleviate the ice formation and recrystallization.

Cryopreservation is an important technique used to store various types of cells, tissues, and organs at very low temperature, usually in liquid nitrogen (−196 °C) [156], and has become crucial in cell biology and regenerative medicine [157,158]. However, cells are not always viable after thawing [145,159]. The freezing and thawing process during cryopreservation causes cryo-injury to cells (Figure 5). Currently, two methods, i.e., slow-freezing [156] and vitrification [160], are commonly adopted in cryopreservation. Prior to addressing the role of AFPs in cryopreservation, we will discuss the association of cryo-injury with freezing with regard to methods other than decreased temperature.

During the slow-freezing process, since the solute concentration inside a cell is higher than that in the medium, the cell is supercooled and ice forms extracellularly [156]. The growth of extracellular ice leaves the unfrozen fraction highly concentrated with salt, leading to dehydration of the cell and destabilization of cellular membranes simultaneously due to osmotic pressure. Incomplete dehydration inside the cell allows intracellular ice formation, which is believed to be detrimental to cells. Eventually the further growth of extracellular ice may cause rupture of the cell membrane. In addition, recrystallization of intracellular and extracellular ice during the thawing process may further damage the cryopreserved cells. Since cell-penetrating CPAs, such as dimethylsulfoxide (DMSO) and glycerol, reduce ice formation by replacing water outside and within the cell as well as stabilize the membranes, the addition of CPAs can increase the post-thaw viability of cryopreserved cells.

Vitrification is a process in which a liquid turns into an amorphous glass solid in the absence of crystallization [160]. Vitrification of cells requires very high concentrations of CPAs and ultrafast cooling rates to completely avoid fatal intracellular and extracellular ice formation [160,161]. In addition to the osmotic stress and chemical toxicity caused by high CPA concentrations, however, vitrification is also associated with ice recrystallization during thawing. In both cases, ice recrystallization during thawing seems to be one of major cold damages. In this context, AFPs are believed to play a crucial role in inhibiting ice recrystallization, improving the cryopreservation efficiency.

### 4.2. AFPs in Cryopreservation

**Figure 6:**
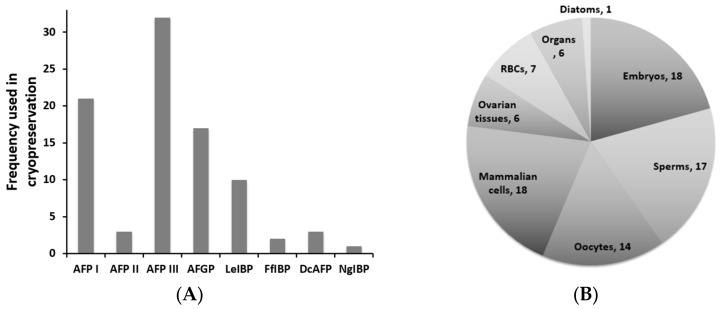
Cryopreservation research using AFPs: (**A**) frequency of AFPs used in cryopreservation; and (**B**) types and frequency of biological samples in cryopreservation using AFPs.

The first application of marine AFPs to the protection of membranes at hypothermic temperatures was made in 1990 using AFGP from Antarctic and Arctic fishes [83]. Since then, marine-derived AFPs have been tested for cryopreservation on numerous occasions. Almost all reports of cryopreservation using AFPs are summarized in Table 1. Of eight AFPs, including nonmarine insect DcAFP, as shown in Figure 6A, type III AFP has been tested most in cryopreservation, followed by type I AFP, AFGP, and LeIBP. This is because type III AFP is easy to produce recombinantly compared with other fish AFPs and because it has been studied longer than other marine-derived AFPs, such as LeIBP and FfIBP. The results listed in Table 1 also showed that hyperactive AFPs do not always ensure better cryopreservation efficiency [65,66,145]. For example, moderately active LeIBP protects mouse ovarian tissue more effectively than 10-fold hyperactive FfIBP [65]. The same result was obtained in human cell line cryopreservation (Hak Jun Kim, unpublished result), consistent with the observation that hyperactive AFPs do not ensure increased IRI activity (Figure 4) [63]. The AFP concentration used in cryopreservations was also determined empirically (Table 1). The IRI endpoint, sometimes expressed as mg/mL, does not indicate the effective amount of AFP in cryopreservation, and the solubility of AFPs and the molar concentration in the freezing medium should also be considered [145]. Quite frequently, higher concentrations of AFPs lead to a decrease in the post-thaw survival of cryopreserved cells, which may be due to the formation of destructive needle-like ice at high AFP concentrations [65,66,68,69,92,162,163].

Cryopreserved biological samples are relatively diverse ranging from diatoms and reproductive cells to embryos and organs (Figure 6B). Most cryopreserved biological samples originated from mammals. Most cryopreservation trials using AFPs have demonstrated that the addition of AFPs could improve post-thaw viability, regardless of the freezing method (slow-freezing or vitrification), storage temperature, and biological sample, but several reports showed no beneficial effects [68,87,88,89,90,91,92,93,94,164,165].

In the cryopreservation of cell lines, AFPs have been used as additives to conventional freezing medium to reduce the high amount of cytotoxic CPAs and reduce freezing damage [31]. Some of the cell types tested for cryopreservation with the addition of AFPs include sperms [167,168,169,170,171,176,178,200], oocytes [66,70,83,177,181,183,185,190,198,199], human liver cells [173], RIN-5F insulin tumor cells [174], diatoms [143], red blood cells [18,144,197], muscle cells [162,179], gut cell [188], islet cells [193], *E. coli* [83], and human cell lines [145] including HeLa cells, NIH/3T3 cells, preosteoblasts (MC3T3-E1 cells), and human ketatinocytes (HaCaT cells). Thus, the addition of AFPs seems to mainly enhance the cryopreservation efficiency regardless of cell type and freezing method, with a handful of exceptions [68,89,90,162,164]. Notably, these exceptions appear to be related to the concentration of AFPs used; indeed, at higher concentrations, AFPs form needle-like ice, which penetrates and destroys cells during freezing [68,143,144,145,162,193,201]. The amount used in cryopreservation also differs between AFPs. LeIBP, which shows lower TH activity, but higher IRI activity than fish AFPs has been used in the range of 0.1–0.8 mg/mL in red blood cells [144], diatoms [143], oocytes [66], and mammalian cell lines [145], whereas fish AFPs have been used at concentrations lower than 0.1 mg/mL, depending on the cell type (Table 1). Interestingly, in the vitrification of mouse oocytes, 0.05 mg/mL FfIBP is more effective at maintaining in murine oocyte quality and embryo development than 0.1 mg/mL LeIBP and 0.1 mg/mL type III AFP [66]. Since the results obtained vary between studies, the utilization of AFPs in cryopreservation needs fine-tuning depending on the type of AFPs, cells, freezing media, and storage temperature.

Embryos from fish [166,186,187,196,202], cows [172,203], sheep [175], rabbits [176], mice [67,92,183,191], and horses [91] were preserved in the presence of AFPs. Early attempts with equine and mouse embryos demonstrated that fish AFPs had negligible effects [91,92]; however, fish embryos subjected to microinjection or incubation in type I AFP solution showed significantly increased survival after chilling at 4 °C or −10 °C. Vitrified 5-somite embryos in type I AFP solution showed similar survival to that of cells recovered from unfrozen embryos [187]. Similarly, AFPs can help improve the survival of embryos preserved at hypothermic temperatures [150,153,154]. These promising results may fuel research in not only hypothermic storage but also vitrification of other embryos such as mammalian embryos.

Lee and colleagues evaluated the beneficial effects of AFPs in vitrification of mouse ovarian tissues [65,67]. Ovarian tissues treated with type III AFPs showed significantly higher intact follicle ratios and lower apoptotic follicle rates than control tissues. The transplanted vitrified-warmed ovaries showed higher intact follicle ratios [65]. In another attempt, all AFP-treated groups had significantly improved follicle preservation with decreasing efficiency in the order of LeIBP > FfIBP > type III AFP [65].

Few studies have evaluated the potential use of AFPs in the hypothermic storage of organs [94,164,184,204]. The TH activity of AFPs has been exploited for subzero preservation of organs. As anticipated, the presence of AFPs decreases cold-induced injury during the hypothermic storage of rat livers [204] and mammalian hearts [69,205] by decreasing the ice formation [189,204,206]. In contrast, Wang et al. reported that higher concentrations of AFGPs have adverse effects on heart preservation [164].

## 5. Conclusions and Perspective

Thanks to their unique properties as biological antifreezes, AFPs have attracted interest from researchers in academia and biomedical fields. In this review, we surveyed the past and current trends in cryopreservation applications of AFPs. The first property of freezing point depression, termed TH, has typically been utilized primarily in the hypothermic storage of tissues and organs. Due to the complexity and size of tissues and organs, more advancement is needed to achieve effective hypothermic storage of these biological materials. The ability to inhibit ice recrystallization is known to neutralize the catastrophic large icy environment for the cryopreserved cells during freezing and/or warming. The third and less characterized function of AFPs is the interaction with cellular membranes and/or integral membrane proteins. It is not likely that these interactions themselves can confer the cryopreserved cells with post-thaw viability. However, AFPs are thought to augment the viability or cryopreservation efficiency of the cells together with the other two features, particularly IRI.

Our physicochemical understanding of unique binding of AFPs to ice crystal has been the main focus of scientists within the last five decades [51,207]. Relatively few studies have evaluated the application of AFPs in cryopreservation. This is mainly because AFPs are expensive to obtain. Therefore, prior and current cryopreservation research has been limited only to moderately active fish AFPs. Additionally, the applications of AFPs has still only partly characterized based on empirical features, similar to other CPAs [31]. In other words, researchers still need to determine the optimal working concentrations of AFPs in cryopreservation; neither TH nor IRI can provide this information.

For the application of AFPs to be practical, a few questions should be addressed. First, mass production of AFPs should be established. Currently, only type III AFP has been produced on the industrial scale owing to its use as an ingredient in ice cream. However, advancements in molecular biology and genomics have improved our ability to produce genes and proteins easily, expanding AFP-related research. Indeed, a mass production system for LeIBP, *Glaciozyma* IBP, has been reported [142]. Additionally, the LeIBP has been shown to yield better post-thaw viability in several studies compared with that of other marine-derived AFPs [65,66,143,144,145]. Second, the behaviors of AFPs in freezing medium should be characterized thoroughly. Typically, freezing medium contains high concentrations of chemicals, such as DMSO, ethylene glycol, polyvinylpyrrolidone, and polyethylene glycol, which may destabilize AFPs, leading to loss of function [208]. Third, functionalized AFPs should be engineered and developed to overcome the limitations of natural counterparts. Mother nature has suggested the use of RD3 and an IBP from Vostok glacial bacterium [137,209]. In both cases, connecting two almost homologous domains increases the TH value cooperatively compared with their monomeric AFPs [139,209]. Studies from the laboratories of Tsuda, Davies, and Holland have demonstrated that the multimerization of native type III AFP can increase TH activity [210,211,212]. Recently, Steven et al. claimed the dendrimer-like AFPs showed higher TH values [213], and Phippen et al. demonstrated 12 AFP-fused protein cage nanoparticles that increased the TH value to more than 50-fold that of monomeric AFP [214]. A few groups have attempted to synthesize AFP or AFGP derivatives to elucidate the underlying mechanism of action and to develop practical applications [215,216,217,218,219,220,221]. Another interesting approach is the development of cell-internalizable or -penetrating AFPs. AFPs are usually nonpenetrating, such that the internal ice formation should be inhibited by high amounts of cytotoxic CPAs. Cell-internalizable AFPs may also reduce the amount of CPAs in freezing medium, eventually increasing the efficiency of cryopreservation.

Finally, it is encouraging that many research groups studying AFP worldwide have started expanding their research into cryopreservation using AFPs. We hope these concerted efforts will accelerate the development of biomedical application of AFPs.

## Figures and Tables

**Table 1:** Lists of AFPs used in cryopreservation of biological samples.

AFPs	Origin Species of AFPs	Cryopreserved Biological Samples		AFP Quantities Used	Freezing Methods	References
		Organisms	Sample Types			
III	Fish	Turbot (*Scophthalmus maximus*)	Embryos	20 nL (final conc. 0.77 mg/mL) of 10 mg/mL type III AFP injected in yolk sac 0.23914 mm^3^)	Vitrification	[166]
I/III	Fish	Gilthead seabream (*Sparus aurata*)	Sperm	1 μg/mL	Vitrification	[167]
I/III/AFGP	Fish	Bull	Sperm	0.1, 1, 10, and 100 μg/mL	Cryopreservation	[168]
LeIBP	Yeast	Boar	Sperm	0.01, 0.1, and 1 mg/mL	Cryopreservation	[169]
III	Fish	Mouse	Ovarian tissue	0.1, 1, and 10 mg/mL	Vitrification	[65]
FfIBP	Bacteria					
LeIBP	Yeast					
AFGP	Fish	Buffalo	Sperm	0.1, 1, and 10 μg/mL	Vitrification	[170]
III	Fish	Buffalo	Sperm	0.01, 0.1, 1, and 10 mg/mL	Cryopreservation	[171]
III	Fish	Bovine	Embryos	10 mg/mL	Hypothermic	[172]
AFGP	Fish	Pig	Oocyte	40 mg/mL	Hypothermic	[83]
I	Winter flounder	Bovine	Oocyte	20 mg/mL	Hypothermic	[70]
II	Sea raven					
III	Eel pout					
III	Notched-fin eelpout	Human	HepG2	2–10 mg/mL	Hypothermic	[173]
I/III/AFGP	Fish	Rat	RIN-5F cells (insulinoma)	10 mg/mL	Hypothermic	[174]
I	Winter flounder	Sheep	Embryo	1 or 10 mg/mL	Hypothermic	[175]
III	Ocean pout					
III	Fish	Rabbit	Sperm	0.1, 1, 10, and 100 μg/mL	Vitrification	[176]
			Embryo	100, 500, and 1000 μg/mL		
III	Fish	Mouse	Oocyte	500 ng/mL	Vitrification	[177]
LeIBP	Yeast	Diatom	Diatom	0.1 mg/mL	Cryopreservation	[143]
AFGP	Fish	Carp	Spermatozoa	2–10 mg/mL	Hypothermic	[178]
I	Fish	Mouse	Pronuclear embryos, 4-cell embryos	0.1 and 1.0 mg/mL	Vitrification	[92]
III				0.1 mg/mL		
I/III	Fish	Sea bream	Spermatozoa	0.1, 1, and 10 μg/mL	Cryopreservation	[165]
AFGP	Fish	Equine	Embryos	20 mg/mL	Hypothermic/Cryopreservation	[91]
DcAFP	Insect	Mouse	A10 smooth muscle cell	1 μg/mL	Cryopreservation	[179]
AFGP	*Gadus morhua*	Mouse	Embryos	20 mg/mL	Vitrification	[180]
III	Fish	Mouse	Ovarian tissue	0, 5, and 20 mg/mL	Vitrification	[67]
III	Fish	Mouse	Mature oocyte	2.5 mg/mL	Vitrification	[181]
I	Fish	Rat	Hippocampal slice cultures	10 mg/mL	Hypothermic	[182]
AFGP	Fish	Pig	Oocyte	40 mg/mL	Vitrification	[183]
AFGP	Fish	Mouse	Embryos	20 mg/mL	Vitrification	[183]
LeIBP	Yeast	Human	Red blood cells	0.4–0.8 mg/mL	Cryopreservation	[144]
III	Fish	Rat	Heart	3, 5, and 15 mg/mL	Hypothermic	[184]
AFGP	Fish	Rat	Cardiomyoctes	0.5–10 mg/mL	Hypothermic (−4 °C)	[162]
III	Fish	Mouse	Oocytes	500 ng/mL	Cryopreservation	[185]
AFGP	Fish	Rat	Cardiac	10 μg/mL, 10 and 15 mg/mL	Hypothermic	[164]
I/III	Fish	Zebra fish	Embryo	40 μg/mL	Hypothermic	[186]
NgIBP	Diatom	Human	Red blood cells	25, 50, and 77 μg/mL	Cryopreservation	[18]
I/III	Fish	Zebra fish	Embryo	40 μg/mL	Vitrification/Cryopreservation	[187]
DcAFP	Insect	Centipede	Gut cells	0.02 mg/mL	Cryopreservation	[188]
III	Fish	Rat	Hearts	15 mg/mL	Hypothermic	[189]
AFGP	Fish	Mouse	Oocytes	1 mg/mL	Cryopreservation	[190]
I/III	Fish	Mouse	Blastocysts	0.1, 1.0 mg/mL	Cryopreservation	[191]
I/III/AFGP	Fish	Mouse	Spermatozoa	1–100 μg/mL	Cryopreservation	[192]
AFGP	Synthetic	Rat	Islet cell	500 μg/mL	Cryopreservation	[193]
I/II/III/AFGP	Fish	Mouse	Oocytes	20 mg/mL	Vitrification	[194]
I	Fish	Human	Myelogenous leukemia cells	0~1000 μg/mL	Cryopreservation	[163]
III	Ocean pout	Chimpanzee (*Pan troglodytes*)	Spermatozoa	1, 10, and 100 μg/mL	Cryopreservation	[195]
I	Fish	Seabream	Embryos	20 nL of 10 mg/mL type I AFP injected	Vitrification	[196]
III	Fish	Turbot	Embryos	10 mg/mL	Hypothermic	[166]
I	Fish	Rat	Liver	1 mg/mL	Hypothermic	[94]
I/III	Fish	Rat	Hearts	10, 15, and 20 mg/mL	Hypothermic	[69]
I/II/III	Fish	Human	Red blood cells	0–1.54 mg/mL	Cryopreservation	[197]
I	Fish	Human	Red blood cells	5–160 μg/mL	Cryopreservation	[68]
III	Eel pout	Mouse	Oocyte	0.1 mg/mL	Vitrification	[66]
FfIBP	Bacteria			0.05 mg/mL		
LeIBP	Yeast			0.1 mg/mL		
III	Eel pout	Bovine	Oocyte	0.5–1 μg/mL	Vitrification	[198]
AFGP8	Fish	Bovine	Oocyte	1 mM (2.6 mg/mL)	Vitrification	[199]
DcAFP	Beetle *Dendroides canadensis*	Buffalo	Semen	0.1, 1.0, and 10 μg/mL	Cryopreservation	[200]
LeIBP	Yeast	Human	Cell lines	0.1 mg/mL	Cryopreservation	[145]
